# A novel multilayer antimicrobial urinary catheter material with antimicrobial properties†

**DOI:** 10.1039/d4ma01045k

**Published:** 2024-12-28

**Authors:** Benjamin Gambrill, Fabrizio Pertusati, Iqbal Shergill, Stephen Hughes, Polina Prokopovich

**Affiliations:** a Cardiff University School of Pharmacy and Pharmaceutical Sciences, Redwood Building King Edward VII Ave Cardiff CF10 3NB UK prokopovichp@cf.ac.uk; b Department of Urology, Wrexham Maelor Hospital Croesnewydd Rd Wrexham LL13 7TD UK; c Maelor Academic Unit of Medical & Surgical Sciences, Gwenfro Buildings Wrexham LL13 7YP UK

## Abstract

Urinary catheters are commonly used in medical practice to drain and monitor urine of patients. However, urinary catheterisation is associated with the risk of developing catheter-associated urinary tract infections (CAUTIs), which can result in life-threatening sepsis that requires antibiotics for treatment. Using the layer-by-layer (LbL) technique, we assembled a multilayer catheter comprising nine quadruple layers (9QL) of alginate, chlorhexidine (CHX), alginate and poly(β-amino ester) (PBAE) built upon an amino-functionalised silicone. The prepared catheter materials were tested for pre-packaged storage conditions and sterilisation techniques. The daily release of CHX was measured at pH 7.4 and pH 5 and simulated urine at 37 °C, which was used to determine the antimicrobial affect. CHX release was detected for a minimum of 14 days in PBS (pH 7.4), pH 5 release media, and simulated urine for the samples tested against storage conditions and sterilisation techniques. Incubation of the prepared material with bacterial cultures for 24 hours restricted bacterial growth compared to incubation with the standard material. The minimum inhibition concentration of CHX for clinically isolated urinary tract infection (UTI) bacterial strains was in the range of 19.4–77.4 µM, at which the released CHX could indirectly prevent bacterial growth for up to 14 days. Based on the daily CHX release from the samples, the hydrolysis of PBAE at pH 5 was gradual, resulting in a greater number of days of preventing bacterial growth, followed by pH 7.4 and then simulated urine. To the best of our knowledge, this is the first report on the use of PBAE in association with a urinary catheter material for the release of an antimicrobial drug.

## Abstract 2

Urinary catheters are commonly used in medical practice to drain and monitor urine of patients.

## Graphical abstract

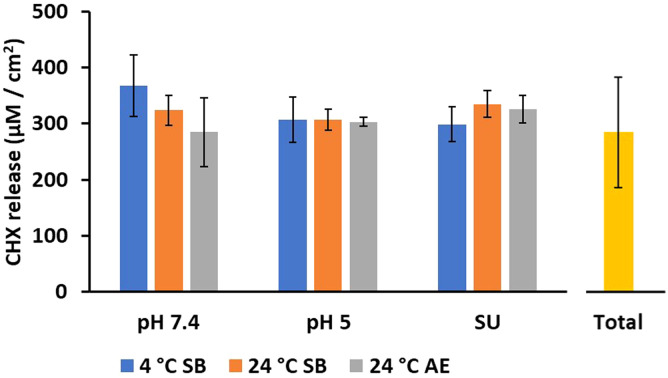

## 1 Introduction

Following hospital admission, many patients require a urinary catheter for urine drainage. In hospitals, 12–25% of hospitalised patients require urinary catheters, making them one of the most commonly used medical devices.^1,2^ The catheter devices are also used intermittently for home self-catheterisation,^3^ as opposed to indwelling which would be used in hospitals, being in place for a longer duration. Both indwelling and intermittent catheterisation involve passage through the urethra to reach the bladder to make urine drainage possible. Consequently, catheterisation is an invasive process, which can result in urethral strictures and subsequent infection.^4^ Those using catheters are more susceptible to urinary tract infections (UTIs), particularly catheter-associated urinary tract infections (CAUTI). Due to the nature of catheterisation, the risk of developing a CAUTI increases between 3% and 10% daily.^5–9^ In hospitals, UTIs are the most common nosocomial infection,^10,11^ where admitted patients would possibly require further tests and treatments, increasing both the length of their hospital stay and healthcare costs, either to the individual or provider.

It is estimated that 30% of nosocomial infections are UTI,^11^ and these infections can be exacerbated when antimicrobial resistance (AMR) is considered. It is believed that by 2050, 10 million deaths will be attributed to multidrug resistance.^12^ At present, it seems that UTIs are treated with antibiotics without considering their causative micro-organisms or antibiotic usage guidelines, which negatively contributes to AMR.^13^ Furthermore, the overuse and incorrect prescription of antibiotics create a selection pressure on the bacteria possessing mechanisms that prevent or limit their susceptibility to antibiotics.^14^ For example, antibiotics using a β-lactam ring in their mechanism of action rely on β-lactam binding to transpeptidase enzymes responsible for peptidoglycan cell wall synthesis.^15^ However, some bacteria produce the β-lactamase enzyme, inactivating the functional part of these antibiotics.^16^ Horizontal gene transfer has been identified to be possible between bacteria species, but also occurs between yeast,^17^ with *Candida* spp. being a common UTI causative fungal microorganism.^18^ Taken together, this data suggests that use of antimicrobials should be better considered, and in the case of UTIs, their causative microorganisms and known resistance should also be considered.

**Fig. 1:**
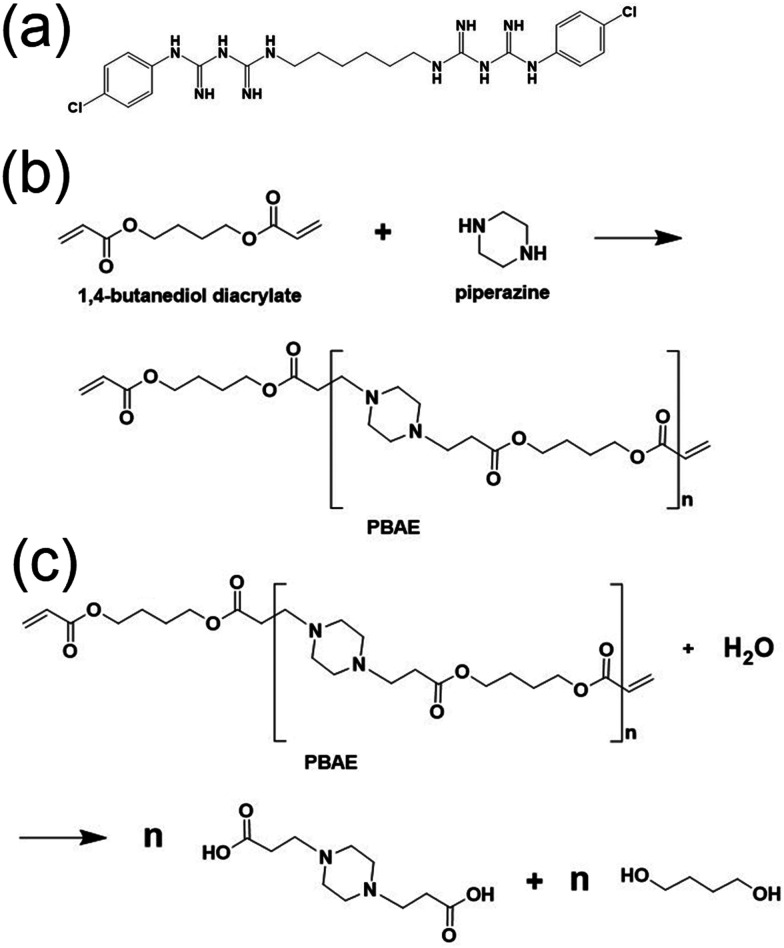
(a) Structure of chlorhexidine.^26^ (b) Poly(β-amino ester) synthesis and (c) hydrolysis. Made using ChemDraw.

The harm caused by use of indwelling Foley catheters (silicone or latex) in 2015 was estimated to reach £2.5 billion with over 2000 deaths in National Health Service hospitals in the UK.^19^ Furthermore, it is believed that around 25% of catheterisations are not necessary,^20^ therefore putting patients at further risk. Therefore, new catheters that drastically reduce, delay, or eliminate the risk of infection are critically needed. To date, the catheters designed to tackle bacterial infection include noble metal-coated catheters (silver, gold, and palladium) and nitrofurazone-containing catheters.^21^ The release of silver ions from the catheter lumen is attractive due to their ability to adhere to bacterial membrane proteins and DNA in the hope of disrupting the functions of replication and cellular integrity.^22^ Nitrofurantoin drugs such as nitrofurazone are employed when the typical UTI drugs (trimethoprim–sulfamethoxazole, cephalosporins, and ampicillins) are made inactive by antimicrobial resistance mechanisms.^23^ However, the recent literature discussed the resistance to nitrofurantoin through mutations in bacterial nitro reductases NfsA and NfsB, resulting in enzymes insensitive to ROS.^24,25^ Considering antibiotic resistance, in this work, we envisaged that the use of the non-antibiotic antimicrobial chlorhexidine (Fig. 1a)^26^ would be highly beneficial against UTIs. Chlorhexidine (CHX) is a broad-spectrum antiseptic and disinfectant, which has been used successfully for oral^27^ and skin antisepsis, and patient bathing in clinical environments.^28^ Specifically in urology, CHX is used clinically to clean urethral tissue prior to catheterisation.^29,30^

This study presents the incorporation of CHX in a catheter material sandwiched between alternatingly charged polyelectrolyte layers deposited on the material surface. Using hydrolysable poly(β-amino esters) (PBAEs), CHX could be gradually released over the usage time between short-term and long-term catheterisation. PBAEs are useful tools to enable gradual drug release, which can be easily synthesised (Fig. 1b) and degraded (Fig. 1c) under physiological conditions.

## 2. Results

### 2.1. PBAE characterisation

GPC was used to determine the degradation of PBAE over time based on the reduction in its molecular weight. The degradation of PBAE indicated that hydrolysis occurs more rapidly at pH 7.4 than at pH 5 (Fig. S2a, ESI†), with PBAE hydrolysis at pH 5 being a more gradual process, as indicated from its molecular weight after 1 and 2 days.

The zeta potential (ZP) values of PBAE during hydrolysis were determined previously (Fig. S2b, ESI†). The measurement of the ZP of PBAE at pH 7.4 and 5 enabled a better understanding of the expected layer interactions when the layers delaminate during the incubation of the samples containing PBAE in release media with a pH of 7.4 and 5. PBAE formed from piperazine and 1,4-butanediol diacrylate was determined to initially have a ZP of approximately +15 mV. In buffer (pH 5) and PBS (pH 7.4), its ZP after day 1 was approximately +7 and +2 mV [mean, *n* = 3], respectively. After 48 hours, the ZP values were approximately −2 (pH 5) and −7 mV (pH 7.4). The charge of PBAE remained negative over a 30-day period, which was approximately −12 mV at both pH 5 and 7.4. The hydrolysis of PBAE and the change in its charge enable drug release as delamination of the layers occurs, as suggested previously.^31,32^ Thus, both the degradation and resulting charge of PBAE demonstrate that it can be useful in layering polyelectrolytes with the aim of releasing drugs. An ideal PBAE would retain its polyelectrolyte charge longer, thus resulting in less delamination, greater drug retention and more sustained drug release. Therefore, choice of PBAE needs to reflect the desired drug release where a quick burst of drug release can be achieved with a quickly hydrolysing PBAE.

### 2.2. Fourier transform infrared spectroscopy

The silicone material was characterised for the presence of functional groups expected to be present in the base layer silicone and silicone amino groups functionalised with APTMS (Fig. S3a, ESI†). The recognised peaks for the silicone base layer include Si–CH_3_ (780, 864, 1280, and 2960 cm^−1^) and Si–O–Si (1000 cm^−1^), corresponding to the silicone material.^33,34^ The peaks associated with amino functionalisation were those demonstrating the addition of amine groups to the surface with N–H bond (1570 and 1640 cm^−1^) and propyl chain C–H bonds (2850–3000 cm^−1^) recognised from the APTMS used for amino functionalisation.^35–37^

The IR spectra were measured for the individual layers built on one another in 1QL to identify the chemical bond signatures indicative of that expected given the composition of the layers (Fig. S3b, ESI†). Identification of the chemical bonds associated with the first layer alginate was difficult with possibilities that the peaks at 3000–3600 cm^−1^ and ∼1095 cm^−1^ could be –OH stretching and C–O stretching, respectively.^38^ Both layers containing CHX (layer 2) and PBAE (layer 4) were expected to have a peak at 1259 cm^−1^ given that both CHX^39^ and PBAE^40^ have C–N stretch from the secondary aromatic amine. Furthermore, the peak at 1259 cm^−1^ was previously assigned to the base layer silicone bending and rocking vibrations of Si–CH_3_.^33,34^

### 2.3. Scanning electron microscopy of base material and 9QL silicone

**Fig. 2:**
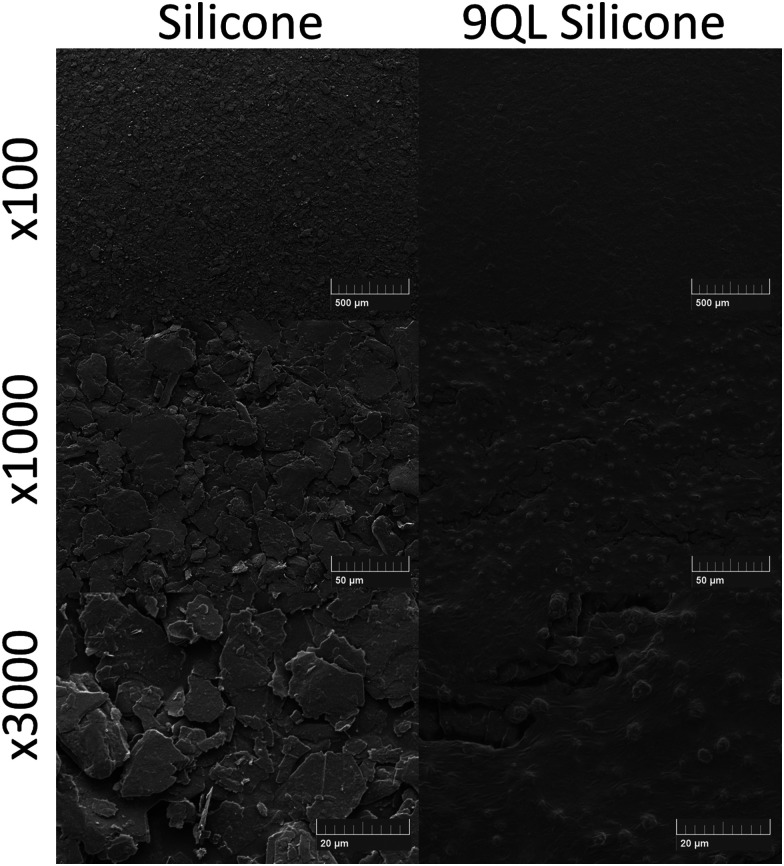
SEM images of base layer silicone and 9QL silicone (bar = 500 µm at ×100, 50 µm at ×1000 and 20 µm at ×3000).

Images of the silicone sample surfaces following LbL assembly to constitute 9QL were taken at magnifications of 100×, 1000× and 3000× (Fig. 2). In this case, nothing appears to be common regarding the surface composition. Images were only taken of one side due to the SEM protocol. It appears that following LbL, the 9QL surface of silicone was smoother than the original silicone material. The surface roughness could be tested using a roughness detector.^41^ A desired material suitable for catheterisation should be as smooth as possible; however, prior to insertion, it is suggested that the catheter and point of entry be lubricated with a suitable water soluble antiseptic or anaesthetic gel.^42,43^ Thus, considering the standard need for using lubricants, it may not be crucial for our design to be as smooth as the standard non-developed material.

### 2.4. CHX release in tested media and storage conditions

**Fig. 3:**
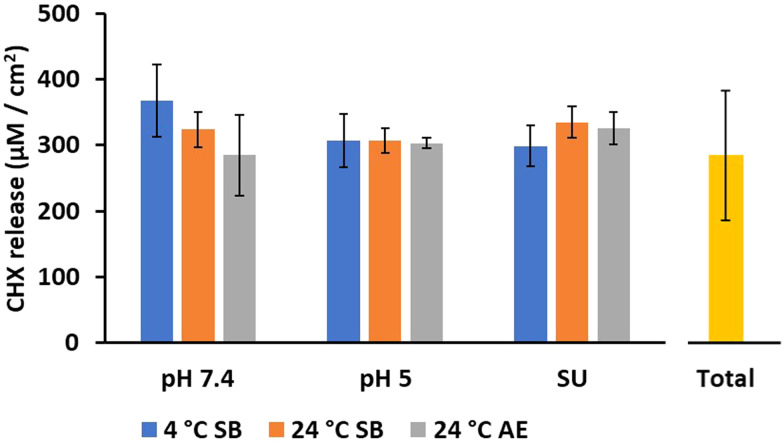
Average CHX released after 14 days for each storage condition and incubation release media. Total represents average total CHX contained in 9QL samples. SB = sealed bag, AE = air exposed [mean ± SD, *n* = 3].

CHX release was evaluated for at least 14 days at pH 7.4, pH 5 and incubation in simulated urine for all three storage conditions tested. The storage conditions and sterilisation techniques were selected based on the expected conditions commercial catheters would be subjected to. Given the degradation of PBAE and required sterility, the catheters were initially stored in the cold (4 °C) in a sealed bag, before removal from cold storage, bag opening and catheter insertion. Using HPLC, it was found that 14 days was the longest duration at which CHX release could be detected for each 24-h sample. The total CHX was measured to be 284 ± 98 µM cm^−2^ when the 9QL samples were incubated in pH 2 media for 7 days at 37 °C. There were no significant differences in the total CHX released after 14 days from any of the silicone samples under their storage conditions. The CHX release after 14 days (Fig. 3) ranged from 285–368 µM cm^−2^ [*n* = 9] for each storage condition and release media tested.

**Fig. 4:**
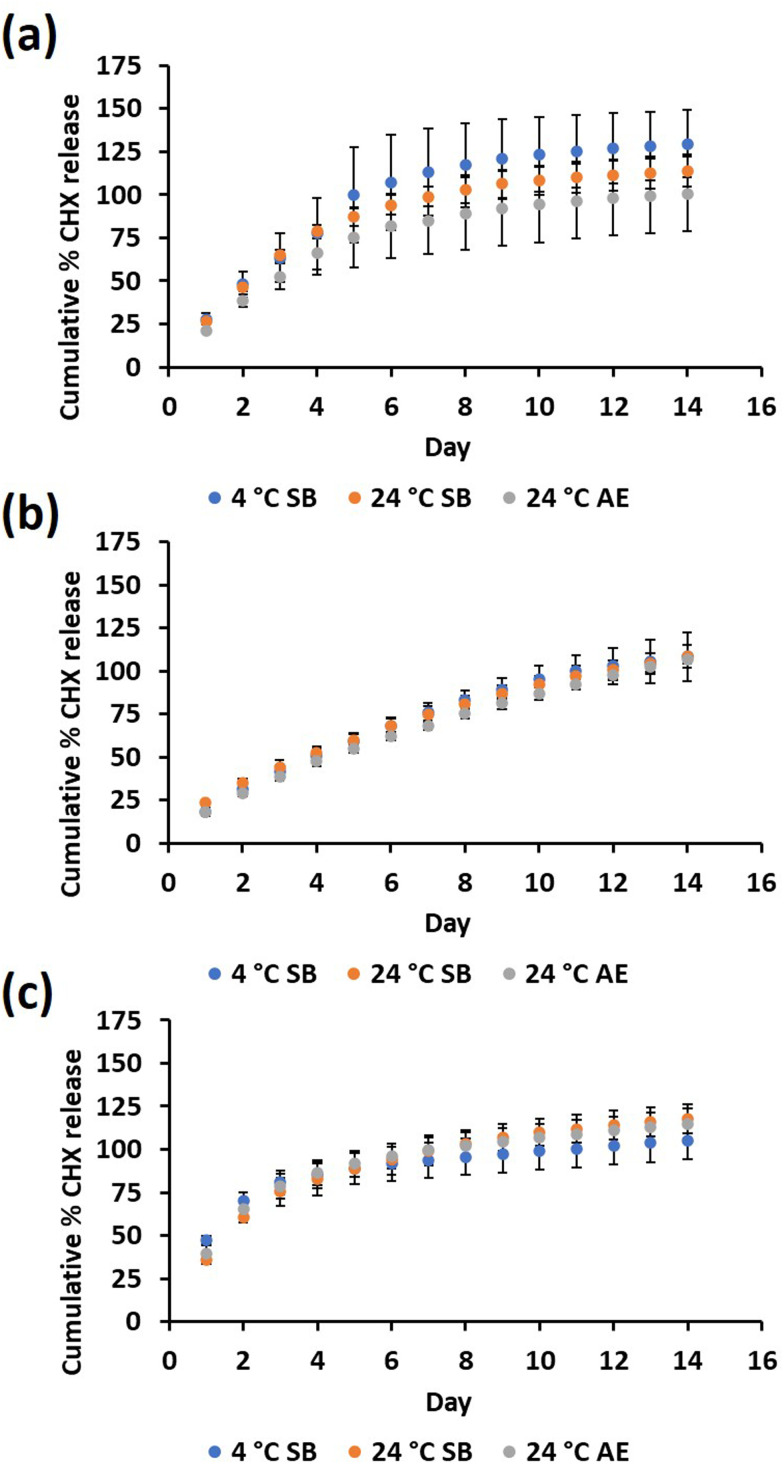
Percentage of CHX release based on average total CHX contained in 9QL samples for each storage condition in (a) pH 7.4 media, (b) pH 5 media and (c) simulated urine. SB = sealed bag, AE = air exposed [mean ± SD, *n* = 3].

According to the CHX release profiles, significant differences were only found among the day 1 release samples. At pH 7.4 (Fig. 4a), the air-exposed samples showed significantly less CHX release than the sealed bag samples stored at 4 °C and 24 °C (*p* < 0.05). At pH 5 (Fig. 4b), the 24 °C sealed bag had significantly more released CHX than both the sealed bag stored at 4 °C and air-exposed samples (*p* < 0.05). In simulated urine (Fig. 4c), the 24 °C sealed bag released less CHX than the sealed bag storage at 4 °C samples (*p* < 0.05). During the first 8 days of cumulative release, there was significantly less CHX released at pH 5. The average CHX release from all the samples regardless of the storage conditions was 326 ± 41 µM cm^−2^ for pH 7.4 incubation [mean ± SD *n* = 9], 306 ± 2.4 µM cm^−2^ for pH 5 incubation [mean ± SD, *n* = 9] and 319 ± 19 µM cm^−2^ for simulated urine incubation [mean ± SD, *n* = 9].

### 2.5. Effect of CHX release when silicone samples were subjected to sterilisation

**Fig. 5:**
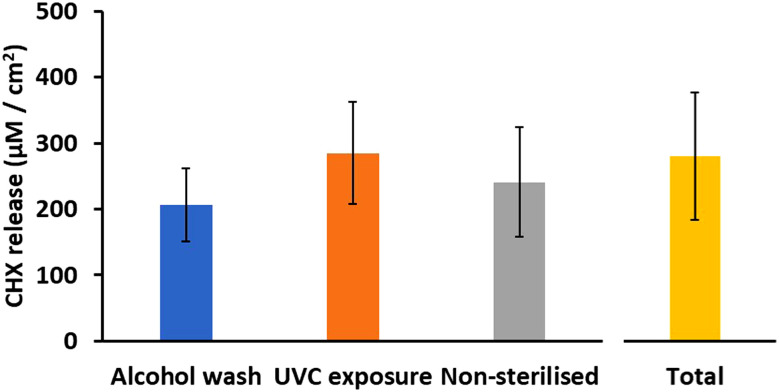
Average CHX released after 14 days for each sterilisation technique. Total represents average total CHX contained in 9QL samples [mean ± SD, *n* = 3].

If a catheter has to be removed and reinserted, sterilisation is required, and thus UVC and alcohol-based sterilisation of the samples were also investigated. There were no significant differences in the CHX release after 14 days following sterilisation. The average cumulative CHX release in PBS (pH 7.4) media after sterilisation (Fig. 5) was 206 ± 56, 285 ± 77 and 241 ± 84 µM cm^−2^ for alcohol wash, UVC exposure and no sterilisation, respectively [mean ± SD *n* = 3].

**Fig. 6:**
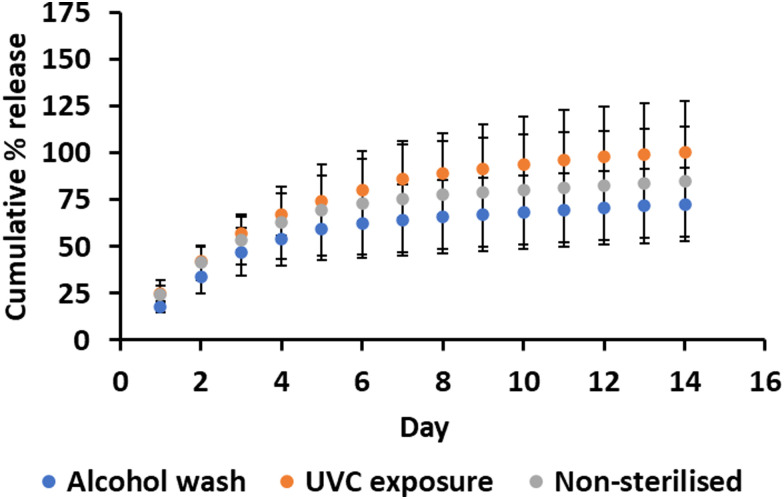
Effect of sterilisation techniques on CHX release [mean ± SD, *n* = 3].

There were no significant differences between the sterilisation techniques in how CHX was released from the samples over the 14-day period (Fig. 6). Alcohol washing slightly reduced the CHX release, which could be attributed to the transesterification reactions between PBAE and ethanol prior to washing with water and incubation in release media.^44^ The reduced CHX release following alcohol washing suggests that this method of sterilisation is unsuitable for this material if catheter sterilisation is required whilst retaining CHX availability. Future work should include further sterilisation after the daily release samples have already been taken to simulate catheter removal, sterilisation, and then re-insertion.

### 2.6. Assessment for planktonic bacterial growth and adherence to 9QL material

**Fig. 7:**
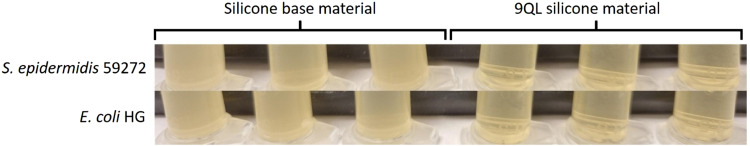
Bacterial culture growth following incubation with base material and 9QL silicone for 24 hours.

Both planktonic bacterial growth during incubation with the 9QL and silicone base material samples and bacterial adherence to the material were used to assess the direct antibacterial effect of the prepared material. After 24 h, the culture of 10^5^ colony forming units (CFU) was visibly capable of further growing to a noticeable extent when incubated with the base material silicone than with the 9QL material (Fig. 7). As expected from the cloudiness of the cultures after 24 h, more planktonic CFU were present when cultures 10^5^ CFU mL^−1^ were incubated with standard silicone than 9QL, as determined by colony counting (Table 1). There were no CFU present at the dilution tested (1 in 10^−6^) for the three *E. coli* HG cultures, suggesting that growth would only be possible up to 10^6^ CFU mL^−1^. Planktonic bacterial growth was prevented to at least one order of magnitude when incubated with the 9QL silicone material instead of the standard silicone material. There were no CFU present for either bacterial species tested following sonication of the material to assess the bacterial adherence on both silicone material types. Thus, this should be further investigated.

**Table 1:** Number of planktonic CFU mL^−1^ when incubated with base material silicone and 9QL silicone

Bacterial species/strain	Average CFU mL^−1^ when incubated with 1 cm^2^ standard silicone (±SD)	Average CFU mL^−1^ when incubated with 1 cm^2^ 9QL silicone (±SD)
*S. epidermidis* 59272	15.7 (±5.7) × 10^7^	0.3 (±0.57) × 10^7^
*E. coli* HG	11.3 (±2.1) × 10^7^	<10^6^

### 2.7. Indirect antibacterial effect of released CHX

The minimum inhibitory concentrations (MICs) for CHX were determined for the same clinical UTI bacterial isolates used previously together with further species/strains (Table 2). The MIC values were in the range of 19.4 to 38.6 µM for *Staphylococcus epidermidis* 59272, *Escherichia coli* HG and *E. coli* H533 [*n* = 6], 38.6 to 77.4 µM for *E. coli* H524, *S. epidermidis* RbG1a and methicillin-resistant *Staphylococcus aureus* (MRSA) 2758 [*n* = 6] and 19.4 to 38.6 µM for *Pseudomonas aeruginosa* LES400. Each of the CHX MIC values found for the bacterial strains, 19.4, 38.6 and 77.4 µM, were compared with the concentration of CHX released each day from the 9QL material to determine which day would be the last day at which bacterial growth would be prevented. This was carried out for all the samples for each storage condition in each release media, and the tested sterilisation techniques (Table 3).

**Table 2:** Bacterial MIC values. The lowest and greatest MIC values determined for each bacterial strain using 96-well plate microdilution approach (*n* = 6). Brackets () show the percentage of samples presenting this MIC value

Bacterial species	Strain	Lowest MIC value (µM)	Greatest MIC value (µM)
*S. epidermidis*	59272	19.4 (16.7)	77.4 (33.3)
*E. coli*	HG	19.4 (33.3)	77.4 (16.7)
*E. coli*	H533	19.4 (16.7)	77.4 (16.7)
*E. coli*	H524	38.6 (33.3)	77.4 (66.6)
*S. epidermidis*	RbG1a	38.6 (83.3)	77.4 (16.7)
MRSA	27258	38.6 (66.6)	77.4 (33.3)
*P. aeruginosa*	LES400	19.4 (33.3)	38.6 (66.6)

**Table 3:** Number of days of expected prevented bacterial growth at each of the MIC values found for each of the bacterial strains (*n* = 3). SB = sealed bag, AE = air expose**d**

	Number of days of expected prevented bacterial growth when MIC is 19.4 µM (±SD)	Number of days of expected prevented bacterial growth when MIC is 38.6 µM (±SD)	Number of days of expected prevented bacterial growth when MIC is 77.4 µM (±SD)
4 °C SB pH 7.4	8.3 ± 1.5	4.7 ± 1.5	4.0 ± 1.7
4 °C SB pH 5	12.0 ± 1.7	8.7 ± 1.2	1.7 ± 0.6
4 °C SB SU	4.7 ± 1.5	3.0 ± 0.0	2.0 ± 0.0
24 °C SB pH 7.4	8.0 ± 1.0	5.0 ± 1.0	3.7 ± 0.6
24 °C SB pH 5	12.3 ± 1.5	5.7 ± 1.5	1.3 ± 0.6
24 °C SB SU	7.7 ± 0.7	4.0 ± 1.0	2.7 ± 0.6
24 °C AE pH 7.4	6.7 ± 1.2	5.3 ± 1.2	3.3 ± 1.2
24 °C AE pH 5	14.0 ± 0.0	6.0 ± 1.7	1.0 ± 0.0
24 °C AE SU	6.7 ± 0.6	4.0 ± 0.0	2.3 ± 0.6
9QL pH 7.4	5.0 ± 2.0	4.3 ± 1.5	2.3 ± 1.5
Alcohol wash pH 7.4	5.0 ± 1.0	3.3 ± 0.6	2.3 ± 1.2
UVC exposure pH 7.4	7.7 ± 3.1	5.0 ± 1.7	2.7 ± 1.2

The smaller the CHX MIC value for the bacterial strain, the greater number of days of which bacterial growth would be prevented, given that the released CHX would be greater than the CHX MIC value. The samples that would enable the longest prevention of bacterial growth would be those exposed to pH 5 media after storage conditions, where bacterial growth would be prevented for between 2 and 12 days. The broader range of days before bacterial growth could be attributed to the more gradual CHX release that was measured at pH 5 and the broader range of MIC values determined. The samples exposed to 4 °C and 25 °C and incubated in buffer of pH 5 would be capable of preventing bacterial growth of *S. epidermidis* 59272, *E. coli* HG and *E. coli* H533 for 14 days given that the MIC of 19.4 µM was always less than that released from the samples. The range of pH values of 38.6 to 77.4 µM for *E. coli* H524, *S. epidermidis* RbG1a and MRSA 2758 would result in lesser days before bacterial growth would be expected with at least 2 days and up to 5 days of prevented growth with this narrower MIC range. In contrast, the MIC values determined for *P. aeruginosa* of 19.4 and 38.6 µM enable for a greater number of days before bacterial growth due to the narrower range between the experimental MIC values.

The antibacterial potential of the samples tested with sterilisation techniques and incubated in PBS (pH 7.4) media was compared with the MIC data the same way as that for the storage conditions. The MIC in the range of 19.4–77.4 µM for bacterial strains resulted in a greater number of days of prevented growth than that with a narrower MIC range, as expected. The samples exposed to the sterilisation techniques had a similar number of days of prevented growth for 9QL silicone and UV exposed samples for the MIC range of 38.6–77.4 µM and 19.4–38.6 µM, with less days for the samples washed with alcohol, where the CHX release was less.

### 2.8. Fourier transform infrared spectroscopy for samples incubated under storage conditions and sterilised

The FTIR spectra for the storage conditions (Fig. S4a, ESI†) appeared unclear in the case of air exposure silicone compared to 9QL silicone and the other conditions. Both samples incubated at 4 °C and non-incubated 9QL have simple FTIR profiles compared to that seen in the 1QL samples (Fig. S3, ESI†), suggesting that the further 8QL deposited represents the same layers after the complete process. Both the 24 °C sealed bag and air exposure samples produced FTIR spectra that do not represent the expected profiles. The FTIR spectral profiles for the sterilisation techniques appear similar compared to the 9QL silicone samples. The sterilised samples (Fig. S4b, ESI†) had similar FTIR spectra to 1QL again, as with the 4 °C incubation and non-incubated 9QL samples. Despite not being able to fully decipher the FTIR spectra of the samples, they could still release CHX and limit planktonic bacterial growth, as demonstrated here.

### 2.9. Scanning electron microscopy of storage and sterilisation images

Images of the silicone sample surfaces were taken at magnifications of 100×, 1000× and 3000× for the storage conditions (Fig. S5a, ESI†) and sterilisation techniques (Fig. S5b, ESI†) tested. There does not appear to be anything in common regarding their surface composition. Images were only taken of one side due to the SEM protocol. It remains unclear from the surface roughness alone how our catheter preparation would perform if scaled up, and therefore further work is needed, for example friction tests with and without the use of lubricants, as previously mentioned.

## 3. Discussion

### 3.1. CHX release is consistent with previous work and not impacted by the expected catheter storage conditions

Generally, the release profiles of CHX at pH 5 and 7.4 are correlated with the PBAE hydrolysis and drug release found in previous similar work.^31,45,46^ Although there were no significant differences in total CHX after 14 days between pH 7.4 and pH 5, in the initial days of CHX release, there was greater release at pH 7.4 than pH 5, which is consistent with the PBAE hydrolysis and ZP measurements of the PBAE at each of the pH values tested. The ZP of the PBAE is crucial for drug release, where delamination is dictated by the environmental pH, and the ZP value exhibited by PBAE. When using PBAE in LbL systems, drug release occurs through both drug diffusion through the layers and drug release based on hydrolysis of PBAE.^47^ Previous work using chitosan (non-hydrolysable) substituted for PBAE demonstrated drug release at both pH 7.4 and pH 5 similar to the drug release profiles at pH 5 using PBAE.^31^ This suggests that the ability of PBAE to be hydrolysed enables greater drug release at pH 7.4, given that PBAE hydrolyses to a greater extent than at pH 5. At pH 7.4, PBAE degradation occurs more rapidly (determined by GPC) and the PBAE charge becomes more negative more rapidly (determined by zeta potential measurements), which elucidates the greater drug release at pH 7.4 in accordance with both methods of drug release, *i.e.*, drug diffusion and delamination by PBAE hydrolysis.

In the preparation of QL, the ZP of alginate prior to the deposition of PBAE is expected to be approximately −30 mV.^46^ The PBAE was added in the LbL process in buffer (pH 5), causing it to exhibit a more prolonged positive ZP over a longer duration. When the samples were tested for drug release, the drug release was greater in PBS (pH 7.4) because the ZP of PBAE deviated from a positive ZP more than that at pH 5. Thus, drug release occurs by delamination due to the weaker electrostatic interactions at pH 7.4 between PBAE and previous alginate layer than in buffer at pH 5. The difference between LbL systems in pH 7.4 and pH 5 has been well studied,^31,45,46^ and here the inclusion of simulated urine as a release medium only helps contextualise the drug release for our desired medical devices. The ZP of PBAE at pH 5 was measured to be approximately 15 and 10 mV for the initial charge and after 24 h, respectively. Alternatively, the initial ZP of PBAE at pH 7.4 was 20 mV, which although greater than that at pH 5, was followed by a more rapid change from positive to negative charge (Fig. S2b, ESI†).

In the development of drug release systems using LbL with a PBAE layer, attention should be placed on the selection of PBAE, specifically its ZP in the expected environmental pH. For example, in a catheter material, immediate drug release is desirable, given the intimacy of catheterisation and the early risk of developing UTI.^48^ In systems where drug release is desirable for longer durations and the quantity of drug release need to be more sustainable, then a PBAE with less rapid degradation is desirable. Thus, further work can include experimental work to generate a broad library of PBAE degradation and ZP changes in accordance with pH changes, enabling users to fine tune the balance between drug release by natural diffusion and layer delamination by PBAE hydrolysis.

The similarity of the cumulative CHX release profiles suggests that incubation under the storage conditions for 30 days did not detrimentally affect the CHX release, thus making the material suitable for use as a catheter. The 30-day incubation period for the samples exposed to air at 24 °C is longer than that desired given the importance of sterility. However, incubation at 4 °C and 24 °C in sterile sealed bags could perhaps be longer than 30-days, which was the period the material was tested here.

At present, it appears that CHX has only been loaded in nanospheres and dip-coated onto a material designed for the fabrication of catheters.^49^ In contrast, here we present CHX directly incorporated using layers of polyelectrolytes, thus avoiding the need to load CHX into nanospheres. CHX was used previously as a filling for catheter balloons, where drug diffusion through the balloon into the bladder resulted in 230 µM CHX release per hour for 72 h.^50^ However, this only represents the catheter balloon, which is responsible for holding the catheter within the bladder, and thus CHX would only be available within the bladder and catheter lumen. In this case, despite the less CHX release in this work, CHX would be available both in the catheter lumen and in contact with the urinary tract using LbL along the catheter length, not only in the catheter balloon. It would be crucial to have CHX available in both the urinary tract and catheter lumen to prevent CAUTI within the urinary tract and encrustation within the catheter lumen.

### 3.2. Potential safety and efficacy of the prepared catheter material

The work presented here does not include the potential cytotoxicity of the CHX, alginate or PBAE present in the material. However, CHX is already widely used in various healthcare products such as antiseptic lubrication gels,^42^ wound bathing^51^ and mouthwash.^52^ A CHX wash containing 1% (2 mM) CHX was suitable in peripartum vaginal wash.^53^ Specific for catheterisation, in studies using 0.1% (2 mM) CHX solution to reduce the CAUTI incidence, there were no reported adverse effects,^30^ suggesting the safety of CHX from a patient point of view. However, in one study reporting the side effects of CHX wash solutions, in 2 patients using 0.5% (10 mM) CHX solution, one developed urethral urticaria and the other vaginal urticaria.^54^ The concentrations used in these washes (2–20 mM) were less than the cumulative range of concentrations released from the material presented here, which is 285 to 368 µM cm^−2^.

In the case of PBAEs, they have been used in gene delivery systems and determined to be safe in several animal models.^55,56^ PBAE is also relatively safe for human tissues owing to its biodegradability and minimal cytotoxicity, both before and after degradation.^57^ Concerns regarding the toxicity of highly cationic charged PBAEs can be resolved by mixing them with anionic polymers,^58^ which is already a feature of our material preparation. Similar to the PBAE degradation and ZP measurements, further work should include the cytotoxicity of PBAEs given the number of possible PBAEs that can be synthesised using amine and diacrylate compounds. Previous cytotoxicity research involving PBAEs used MTT and LDH assays, which should be employed according to ISO 10993-5 requirements, given that all medical devices are required to conform with these requirements.

Finally, biodegradable alginate oligosaccharides have been shown to have no cytotoxicity against mammalian cells using animal models^59^ and used as ureteral stents with comparable cytotoxicity to standard stents.^60^

Despite this, further work should include specific testing of the release media containing CHX, alginate and PBAE against urological human cell lines. It would be expected that the greatest concentration of these three released components would be released in the initial day of incubation, and this would be where cytotoxicity testing would be the most crucial.

### 3.3. Daily CHX release provides antibacterial effect for up to several days

It appears that CHX release because of PBAE hydrolysis is crucial in the number of days at which bacterial growth would be prevented, for example, pH 5 results in more gradual PBAE hydrolysis with subsequent gradual release of CHX. This gradual CHX release, most often greater than the lowest MIC value of 19.4 µM found for some bacterial strains, resulted in the maximum number of days of preventing growth. In contrast, the samples incubated in simulated urine were found to have the most rapid CHX release in the initial days with remarkably less CHX released in the following days. This resulted in overall less days before bacterial growth occurred because the initial days had a greater CHX release than other release medium.

The current gold standard urinary catheters used by healthcare institutions are noble metal nanoparticle-coated catheters, such as that produced by Bard Inc. (USA).^61^ Cohort analysis suggest these catheters can significantly reduce the risk of patients developing bacteriuria, defined as the presence of bacteria of ≥10^5^ CFU mL^−1^.^62^ Thus, any new catheters devised should be compared with noble metal nanoparticle-coated catheters to determine if nanoparticle release has a greater antibacterial effect than, in the present case, the CHX release.

Many studies have been reported on reducing the chance of developing CAUTI with the daily increased risk. For example, 67% of catheterised patients had CAUTI,^63^ with the risk of developing CAUTI increasing up to 10% daily.^5–9^ However, the increasing risk of CAUTI each day cannot be matched with CHX release because CHX released from the material decreases each day whilst CAUTI risk increases. Thus, to compete with the increasing risk of developing CAUTI, a more active drug delivery system would be required, such as the direct delivery of drugs to the urinary tract, such as *via* a balloon in a three-way catheter. Usually, the third channel of a catheter is used for bladder irrigation following surgery^64^ but this can be repurposed for the delivery of drugs, which can increase in concentration daily to match the increasing risk of developing CAUTI. Thus, it can be desirable to have the catheter design proposed here be developed as a three-way catheter to not only enable the release of CHX from the material within the urethra, but also in the bladder delivered by a separate channel.

This work did not specifically consider biofilm formation on the material given that our aim was to prevent bacteria adherence from occurring. In this case, the aim of this work was to prevent biofilm establishment given that encrustation on the outside of the material can be painful for patients, while encrustation on the inside of the catheter can cause catheter blockages.^65^ Here, we show that the 9QL material limited planktonic bacterial growth compared to the normal silicone material, which has potential for preventing adherence. However, although we tested strains of *E. coli*, *S. epidermidis*, MRSA and *P. aeruginosa*, other biofilm forming species should be tested for sensitivity to CHX, such as *Klebsiella* spp. and *Proteus* spp., given that preventing the survival of these species will be very important in preventing biofilm formation through bacterial adherance.^66^ Furthermore, it can be suggested that the use of fungi *Candida* spp. will be crucial to studying CAUTI as a pathogen,^18^ for which CHX should be suitable given its role as an antimicrobial not just an antibacterial agent.

## 4. Experimental

### 4.1. Equipment and materials

Piperazine, 1,4-butanediol diacrylate, dichloromethane, diethyl ether, 1-hexanol, cyclohexane, 3-aminopropyl trimethoxysilane (APTMS), chlorhexidine, potassium chloride, hydrochloric acid, BHI broth powder and BHI agar powder were purchased from Merck (NJ, USA). Sodium acetate trihydrate, acetic acid, Triton X-100, ammonium hydroxide solution, sodium alginate, ethanol, PBS tablets and acetonitrile (ACN) were purchased from ThermoFisher Scientific, (MA, USA). Polyethylene glycol standards were purchased from Honeywell (NC, USA) and Malvern Panalytical (UK). Silicone material was purchased from Goodfellow (UK). The bacterial isolate strains used were *Escherichia coli* strains HG, H533 and H524, *Staphylococcus epidermidis* strains 59272 and RbG1a, MRSA 27258, and *Pseudomonas aeruginosa* LES400.

Centrifugation was carried out using a Sigma 3-16KL from Merck. Gel permeation chromatography (GPC) and high-performance liquid chromatography (HPLC) were carried out using devices from Shimadzu, UK. The GPC column used was Superdex 75 10/3000 GL purchased from Cytiva, MA, USA. Fourier transform infra-red (FTIR) spectroscopy was carried out using a Cary 600 FTIR spectrometer and data recorded using the Resolutions Pro programme by Agilent, CA, USA. The HPLC column used was a Zorbax Eclipse column, XDB-C8 4.6 × 150 mm 5 µM, which also produced by Agilent. Scanning electron microscopy (SEM) was carried out using a Desk Gold Sputter by Torontech, Ontario, Canada, and images taken using a Tescan Vega SEM from Brno, Czech Republic. The UV-C (254 nm) light source used for UV irradiation was purchased from Labortechnik, Germany.

### 4.2. Poly(β-amino ester) synthesis and characterisation

A mixture comprised of 379 mg (4.4 mmol) piperazine, 793 mg (4.0 mmol) 1,4-butanediol diacrylate and 5 mL dichloromethane was incubated at 50 °C for 48 h. After incubation, the mixture was added to 30 mL diethyl ether and subjected to centrifugation for 10 min at 13 300 rpm. Following centrifugation, the PBAE precipitated and excess solvent was poured off and the resulting mixture was dried at room temperature (24 °C) for 24 h.

For PBAE degradation by hydrolysis, 10 mg mL^−1^ PBAE was dissolved in PBS (pH 7.4) and 0.1 M sodium acetate buffer (pH 5). Sodium acetate buffer (1 L) was prepared using 9.52 g sodium acetate trihydrate and 1.74 mL acetic acid. Sodium acetate pH 5 buffer was used as the mobile phase during GPC, where the samples were passed through the column to determine their molecular weight. Immediately, 0.5 mL from the dissolved PBAE sample was taken and every 24 h a 0.5 mL sample was taken from the dissolved PBAE sample being incubated at 37 °C. The molecular weight was determined using polyethylene glycol standards of known molecular weight and their time of elution from the column to produce a standard curve (data not shown). The ZP of PBAE was determined previously^67^ with 20 mg mL^−1^ PBAE dissolved in PBS (pH 7.4) and sodium acetate buffer (pH 5) using a folded capillary zeta cell.

### 4.3. Silicone amino functionalisation and layer-by-layer process

The silicone samples were prepared using the layer-by-layer (LbL) deposition technique^31,46,68^ on amino-functionalised medical grade silicone. For amino-functionalisation, 16 mL 1-hexanol, 75 mL cyclohexane, 4.8 mL diH_2_O and 17.7 g Triton X-100 were mixed and stirred. Once colourless, 600 µL ammonium hydroxide solution was added, followed by stirring for 20 min. 1 cm^2^ squares of silicone and 50 µL APTMS were added to the mixture and incubated at 24 °C for 24 h.

The LbL solutions used were 2 mg mL^−1^ sodium alginate, 10 mg mL^−1^ chlorhexidine, and 2 mg mL^−1^ prepared PBAE, all prepared in 0.1 M sodium acetate buffer (pH 5). Sequentially, the amino-functionalised silicone samples were submerged in the sodium alginate solution for 10 min with frequent agitation, and then the samples were dipped in wash buffer (0.1 M sodium acetate buffer pH 5), next they were submerged in chlorhexidine solution for 10 min, followed by wash buffer, sodium alginate solution for 10 min, wash buffer and PBAE solution for 10 min, and then final wash buffer, all resulting in 1 quadruple layer (1QL) being deposited on the material. Then 8 more quadruple layers were added to the silicone samples, resulting in a total of 9 quadruple layers (9QL). The prepared samples of layered silicone were dried at 24 °C for 24 h.

### 4.4. FTIR spectroscopy and scanning electron microscopy

The samples used were the base silicone material, functionalised silicone, silicone with CHX incorporated and silicone with PBAE incorporated. Absorbance spectra were produced using 64 scans at a resolution of 8 cm^−1^ per sample, with a scan range of 3500–600 cm^−1^. The 9QL samples after storage condition incubation were also investigated by FTIR. Both sides of each sample were scanned by FTIR and the raw data averaged to represent the entire silicone sample.

The silicone samples were coated with ∼50 nm layer of gold and multiple SEM images were taken for each sample at magnifications of ×100, ×1000 and ×3000.

### 4.5. Storage conditions and sterilisation

Silicone samples of 1 cm^2^ were prepared as before with 9QLs using LbL and the samples dried overnight before testing the effect of storage conditions. The storage conditions tested were chosen to mimic that of catheter devices before use, as follows: (i) placed in a sealed plastic bag at 4 °C, (ii) placed in a sealed plastic bag at 24 °C and (iii) open exposure to air at 24 °C, each for 30 days. Three samples were incubated under each of the storage conditions.

For the alcohol sterilisation of medical equipment,^69^ samples of 9QL silicone were dipped and agitated in a solution comprised of 70% ethanol and 30% diH_2_O for 2 min before being left to rest in ethanol for 2 min. The samples were removed from ethanol, agitated to remove the fluid, dipped in ddH_2_O for agitation and left to rest for 2 min each. Once alcohol had been washed off the samples, they were removed and agitated again to remove the fluid. In the case of UV irradiation based on UV-C sterilisation bags,^70^ samples of 9QL silicone were incubated under UV-C (254 nm) 1 cm away from the light source, for 3 min for each face of the silicone samples. The samples were flipped over to expose their other side and again irradiated for 3 min. After storage, the samples were also imaged using SEM and scanned using FTIR.

### 4.6. CHX release characteristics

To test the CHX release from multi-layered silicone after incubation under the different storage conditions, the silicone samples were placed in either PBS (pH 7.4), 0.1 M sodium acetate buffer (pH 5) or simulated urine (Table S1, ESI†), each 1 mL, incubated at 37 °C and replaced every 24 h. PBS was prepared using tablets and sodium acetate buffer prepared as previously stated. The collected release media was tested for CHX using HPLC based on a calibration curve using known CHX concentrations. The mobile phase for HPLC was isocratic, consisting of 70% sodium acetate pH 5 buffer (as prepared previously) and 30% ACN. The HPLC runs were 15 min with the detection wavelength at 239 nm for CHX and sample injection volume of 20 µL. Samples were taken daily for 14 days, where the peaks attributed to the presence of CHX were visible based on previous work (unpublished) determining the maximum duration at which CHX was released.

To determine the total drug content, three 9QL silicone samples were incubated at 37 °C for 7 days in hydrochloric acid-potassium chloride buffer pH 2 (0.745 g potassium chloride and 0.0772 g hydrochloric acid in 100 mL diH_2_O) and the resulting sample drug content was measured using HPLC as above.^31^ Due to the pH of the incubation buffer, the samples were diluted 1 : 1 in PBS (pH 7.4) to make them appropriate for use in HPLC.

The concentration of CHX released was converted to µM cm^−2^ in accordance with the surface area to volume ratio (2 cm^2^ mL^−1^) of placing a double-sided 1 cm^2^ material modified by LbL sample in 1 mL of release media.

### 4.7. Statistical analyses

The average daily CHX release in each of the release media was compared across the storage conditions and sterilisation techniques tested for each day. This was to determine if the tested conditions affected the CHX release given that differences in CHX release in each release media were already established.^45,46^

Significance differences across the conditions were tested using analysis of variance test (ANOVA) and Tukey's test. The test outcomes were considered statistically significant if *p* < 0.05.

### 4.8. Assessment for planktonic bacterial growth and adherence to 9QL material

Clinical bacterial isolate strains from the urinary tract were spread onto BHI agar plates, incubated overnight at 37 °C and colonies picked into 10 mL BHI broth, and incubated for 24 h at 37 °C, producing cultures of approximately 10^8^ CFU mL^−1^. Subsequently, the cultures were diluted 1 : 1000 in BHI broth, producing cultures comprised of approximately 10^5^ CFU mL^−1^.

The silicone materials (1 cm^2^), both base material and 9QL, were incubated with 1 mL of bacterial culture comprised of 10^5^ CFU mL^−1^ for 24 h at 37 °C in wells of a 24-well plate. The resulting bacterial culture grown (planktonic cells) in the presence of the different types of silicone material was retained. Bacterial adherence, as a result of incubation with bacterial culture grown for 24 h, was determined by taking 1 cm^2^ samples and lightly agitating them in sterile PBS to remove non-adhered bacteria. To remove the adhered bacteria cells the samples, they were placed in microcentrifuge tubes containing sterile PBS and sonicated for 5 min. The concentration (CFU mL^−1^) of planktonic and adhered bacterial cells was estimated using diluted cultures (×10^−6^) and plating on BHI agar plates incubated for 24 h at 37 °C.

### 4.9. Indirect antibacterial effect testing of 9QL material

CHX (5 mg mL^−1^) was dissolved in PBS and 100 µL pipetted into the first column of a 96-well plate. In the other wells, 50 µL PBS was added. Next, 50 µL from the initial column well was removed and placed in the next column, mixed and 50 µL retained in the pipette, which was carried out across the 96-well plate, and the final 50 µL retained in the pipette was discarded, and thus every well had 50 µL of CHX solution. Following the addition of 100 µL diluted (10^5^ CFU mL^−1^) bacterial culture, the final CHX concentrations tested were approximately 4946.1, 2473.1, 1236.5, 618.3, 309.2, 154.5, 77.4, 38.6, 19.4, 9.7 and 4.7 µM. The final well in each row was comprised of 100 µL PBS and 100 µL diluted bacterial culture, serving as a positive control. The plates were prepared to collect six MIC values for each bacterial strain and the plates were incubated at 37 °C for 24 h. The MIC value was determined as the last well before that which appeared similar to the positive control where bacterial growth occurred.

The expected day before bacterial growth was determined as the last day where the released CHX concentration was less than the independently determined MIC value for each bacterial strain. Both the lowest and highest MIC values for each strain were used to determine the range at which bacterial growth would be prevented.

## 5. Conclusions

This research demonstrated that a whole catheter device of our design would be sufficient for use in medical environments given that our design was unaffected by the catheter storage and sterilisation techniques. The CHX release was capable of directly preventing bacterial growth compared to the standard material for planktonic cells, as determined by both visible culture growth and dilution plating. Given that there was no indication of bacterial adherence to either material type at the bacterial culture dilution tested, this provides opportunities for further research to expand on this material as a potential candidate for the fabrication of medical devices. Other required studies would be an assessment of material safety from both cytotoxicity and patient comfort points of view. This work demonstrated that LbL preparation can be used on a material commonly used in urinary catheters, enabling CHX release from the material measured for at least 14 days, and antibacterial effect for up to 14 days.

## Data availability

The data related to this study are not publicly available due to ongoing extended research work. The data are available from the corresponding author upon reasonable request.

## Conflicts of interest

The authors declare no conflicts of interest.

## Supplementary Material

MA-006-D4MA01045K-s001
