# Supplementary material for: A novel multilayer antimicrobial urinary catheter material with antimicrobial properties
Source: Mater Adv. 2024 Dec 28;6(3):1020–30. doi: 10.1039/d4ma01045k (PMC11701715; doi:10.1039/d4ma01045k)
Supplement: MA-006-D4MA01045K-s001 [file MA-006-D4MA01045K-s001.pdf]

Supplementary Information (SI) for Materials Advances.
This journal is © The Royal Society of Chemistry 2024

Table S1. Synthetic urine composition

**Compound**	**g/L**	**mM**
$CaCl_2$	0.65	4.40
$MgCl_2$	0.65	3.20
NaCl	4.60	78.70
Na_2SO4	2.30	16.20
Na_3citrate	0.65	2.60
$Na_2(COO)_2$	0.02	0.15
KH_2PO_4	4.20	30.90
KCl	1.60	21.50
NH_4Cl	1.00	18.70
$CO(NH_2)_2$	25.00	417.00

[a] Merck, UK, [b] VWR, PA, USA, [c] Honeywell, NC, USA.

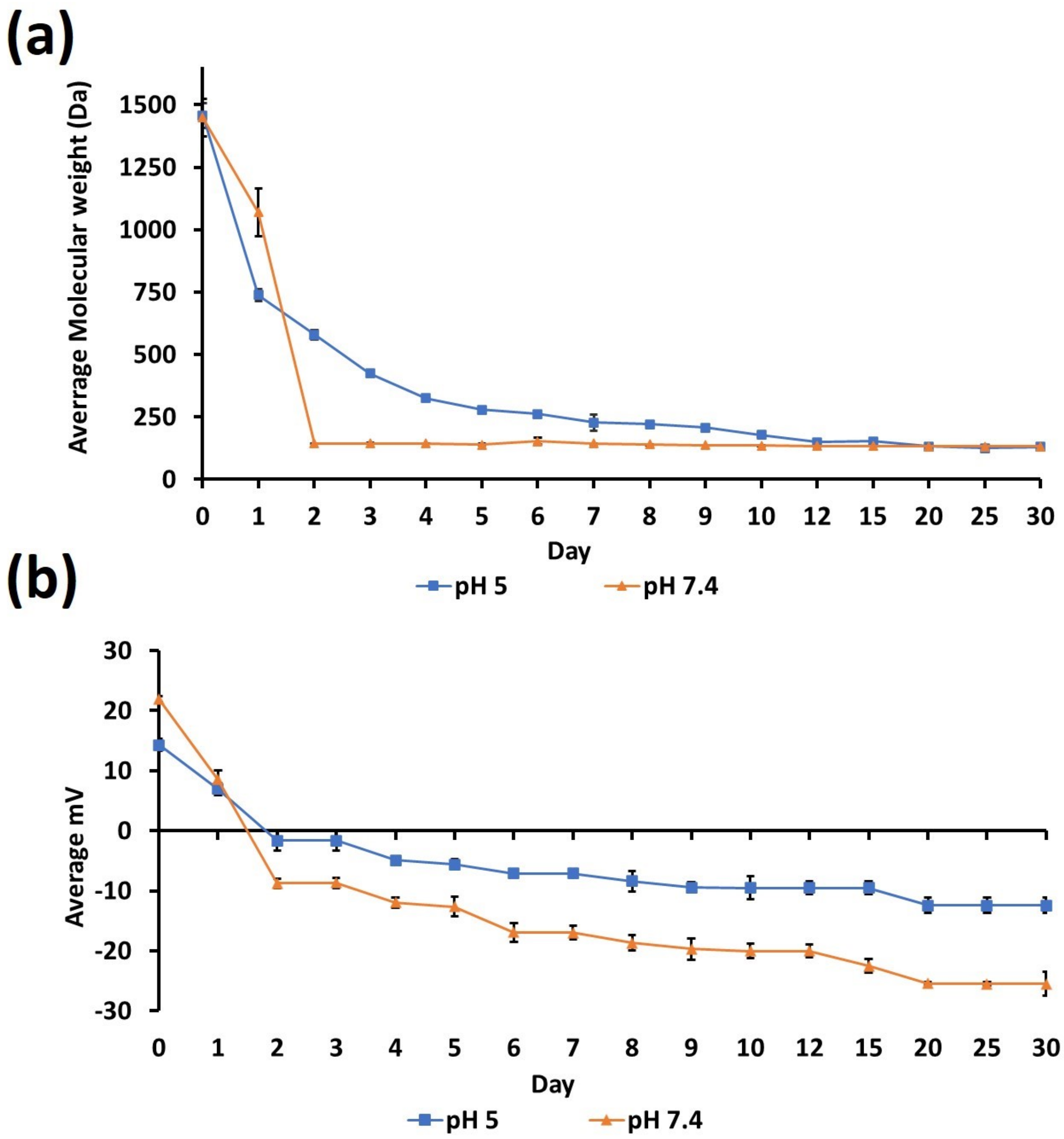

Figure S2. Polymer characteristics (a) Poly β-amino ester hydrolysis and (b) zeta potential determination [mean, n = 2].

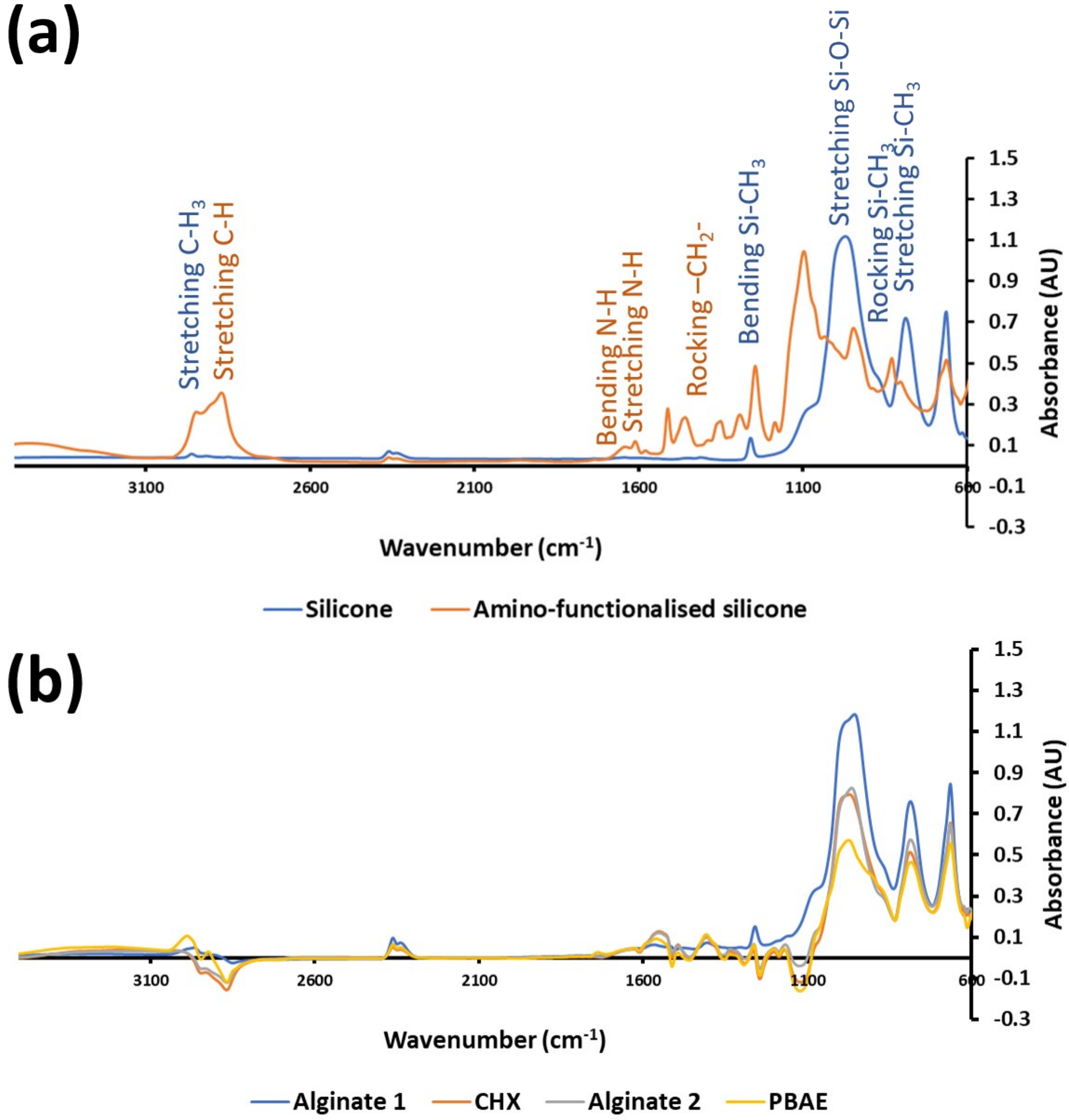

Figure S3. FTIR spectra (a) silicone material and amino-functionalisation, and (b) layers comprising 1QL [n = 2, each side of silicone sample].

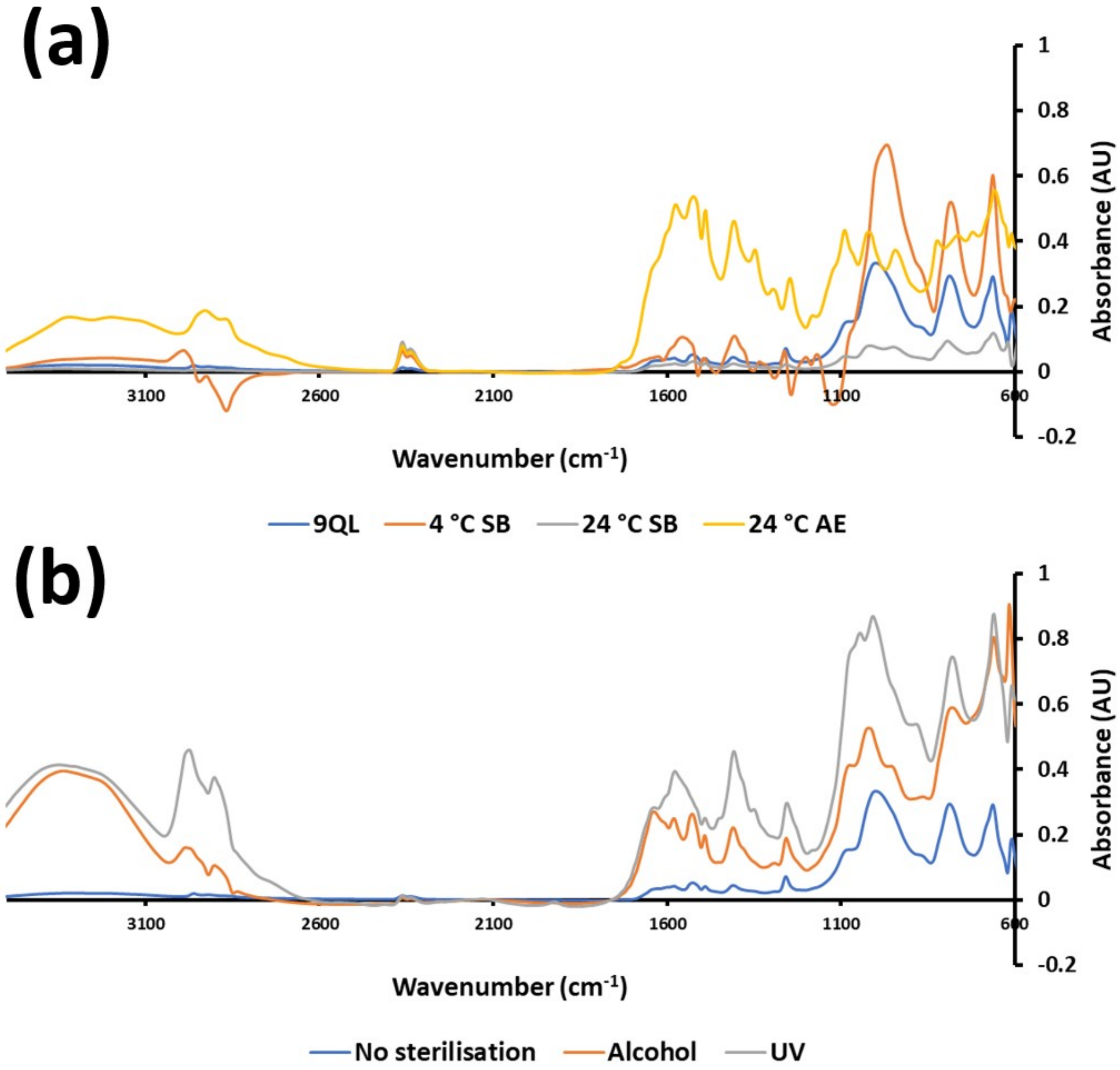

Figure S4. FTIR spectra following material preparation testing (a) storage conditions and (b) sterilisation techniques. SB = Sealed bag, AE = Air exposed [n = 2, each side of silicone sample].

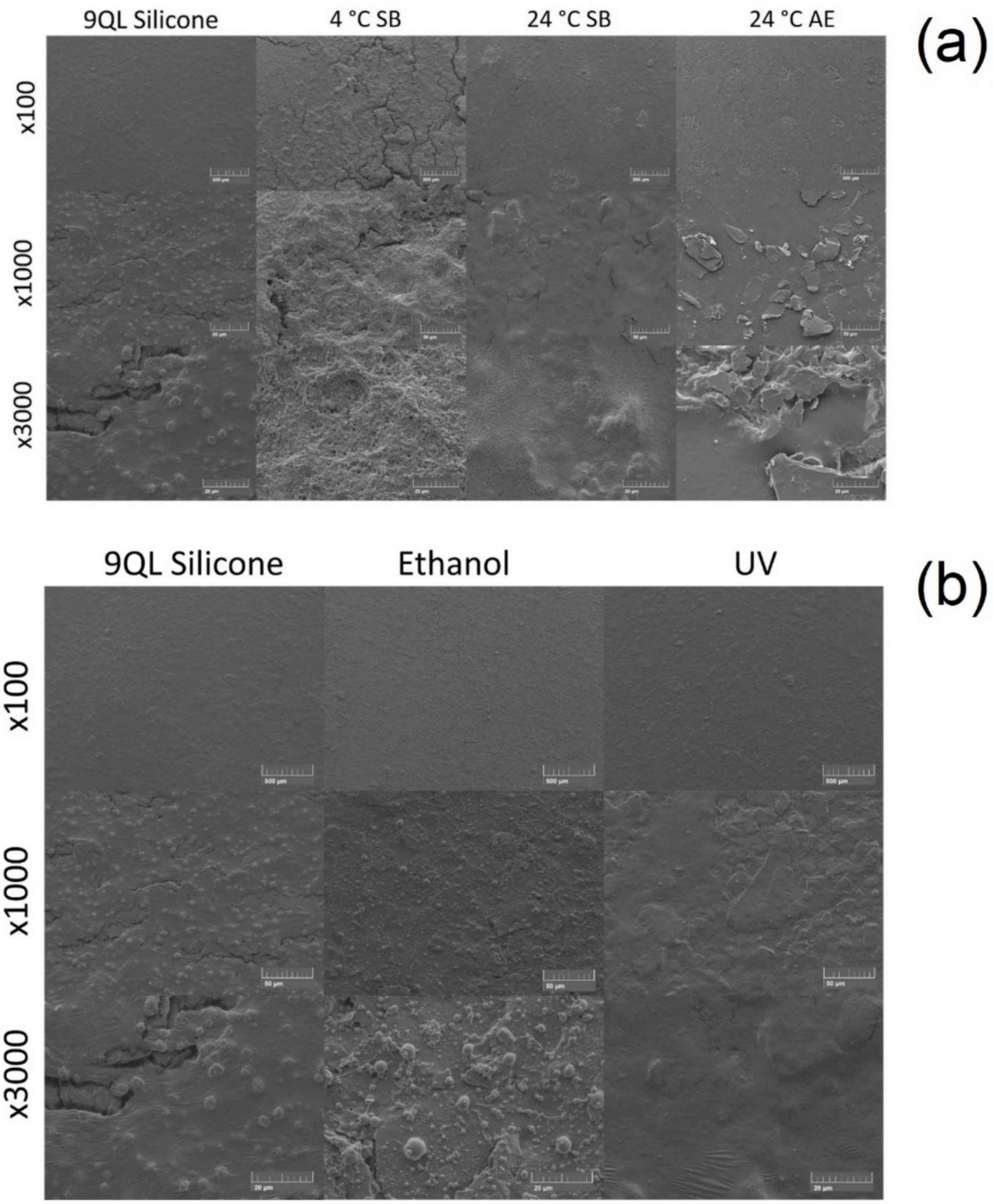

Figure S5. SEM images of material surfaces after incubation at storage conditions for 30 days, compared with 9QL silicone (a) and SEM images of material surfaces sterilised with ethanol and irradiated with UV, compared with 9QL silicone (b). bar = 500 μm at x100, 50 μm at x1000 and 20 μm at x3000. SB = Sealed bag, AE = Air exposed
